# Medication overuse headache: a review of current evidence and management strategies

**DOI:** 10.3389/fpain.2023.1194134

**Published:** 2023-08-08

**Authors:** Yabets Tesfaye Kebede, Bekri Delil Mohammed, Beimnet Ayenew Tamene, Abel Tezera Abebe, Rabbi Waqshum Dhugasa

**Affiliations:** ^1^ School of Medicine, Faculty of Medical Sciences, Institute of Health, Jimma University, Jimma, Ethiopia; ^2^ Department of Nursing, Adama General Hospital and Medical College, Adama, Ethiopia

**Keywords:** medication overuse headache, migraine headache, headache chronification, managment, symptoms, diagnosis

## Abstract

The third edition of the International Classification of Headache Disorders (ICHD-3) defines medication-overuse headache (MOH) as a headache that develops when a person regularly uses acute or symptomatic headache medications excessively (10 or more, or 15 or more days per month, depending on the medication) for a period of time longer than 3 months. Even though it may not be reported as frequently as it actually is, it affects about 5% of the general population on average. It typically happens following repeated anti-pain medication use for pre-existing headache disorders, such as migraines. Anti-pains can also be used frequently in patients with pre-existing headache disorders for reasons other than treating headaches, such as psychological drug attachment. MOH is linked to a number of illnesses, such as anxiety, depression, and obsessive compulsive disorder (OCD). Both simple and complex types are possible. Additionally, there is no universal consensus on how to treat MOH, but drug discontinuation is the best course of action. Using the medical subject headings “Medication Overuse Headache,” “Migraine Headache,” “Tension Headache,” “Chronification of Headache,” and “Antipains,” an all-language literature search was done on PubMed, Google Scholar, and Medline up until March 2023. We looked into the epidemiology, risk factors, pathophysiology, clinical characteristics, comorbidities, diagnosis, management, and preventative measures of MOH in the literature. This article focuses on the MOH research themes.

## Introduction

Medication overuse headache (MOH) is a common, challenging, and disabling condition that affects millions of people worldwide. MOH occurs when people who suffer from primary headaches, such as migraines or tension-type headaches, use painkillers too frequently or in excessive doses. This paradoxically leads to a worsening of headache frequency and severity. MOH can have a significant impact on the quality of life and productivity of the affected individuals, as well as a high economic burden for the health care system.

The aim of this article is to provide an overview of MOH, including its definition, epidemiology, risk factors, pathophysiology, symptoms, diagnosis, treatment, and prevention. We will also discuss some of the challenges and controversies surrounding MOH, such as the role of different types of medication, the optimal duration and method of withdrawal, and the effectiveness of preventive therapies. We hope that this article will help health care providers and patients recognize and manage MOH in a timely and appropriate manner.

## Epidemiology

MOH is estimated to affect approximately 63 million people (1). According to many studies, the prevalence of MOH in the general population ranges between 0.5% and 7.2% (2–4). Higher rates have been reported in Russia (7.6%) (5). Among patients at specialized headache centers, the prevalence of MOH ranges from 30% to 50%, which is much higher than in the general population (6).

The prevalence of chronic migraine in children and adolescents in the United States was found to be 0.79% when medication overuse was excluded and 1.75% when it was included. MOH criteria were met by 21%–52% of pediatric patients with chronic headaches. MOH prevalence in the pediatric population ranges from 0.3% in Taiwan to 3.3% in Italy, with others falling somewhere in between (6–8).

Most studies show that MOH is more common in females, with a male-to-female ratio of around 1: 3–4, and it is more common in middle-aged adults aged 30–50 (2, 9, 10).

According to multiple headache center studies on elderly populations, 30%–35% of patients over the age of 64 overuse medications (11, 12). Despite this, the overall prevalence of MOH decreases among the elderly. In Taiwan, the prevalence was 1.0% among those aged 65 and above (13).

Concerning MOH in specific ethnic groups and minorities, a European study found an increased prevalence of MOH in first-generation migrants, with socioeconomic, cultural, and genetic factors being the main causes (14).

## Risk factors

Migraine is the most common risk factor associated with MOH, affecting 78% of patients. Other primary headache disorders, such as tension-type headaches or cluster headaches, can also result in MOH (15).

Several studies have found that patients who overuse analgesics for non-cephalic chronic medical conditions such as rheumatoid arthritis or ulcerative colitis are much less likely to develop MOH unless they have a history of primary headaches, specifically migraine (16, 17).

According to Zwart et al.'s population-based 11-year follow-up study, individuals who used analgesics daily or weekly at baseline had a higher risk of developing MOH, especially among those with chronic migraine (18).

A more recent large prospective population-based study by Hagen et al., which included over 25,000 patients who did not have chronic daily headaches at baseline but developed MOH 11 years later, identified additional risk factors for MOH in people with chronic headaches. According to this study, those who used tranquilizers on a regular basis or who had a combination of chronic musculoskeletal complaints and gastrointestinal complaints and a Hospital Anxiety and Depression Scale score of 11 or more had a 5-fold risk of developing MOH. The study goes on to explain how physical inactivity (defined as less than 3 h of hard physical activity per week) and smoking (daily vs. never) are risk factors for MOH (19).

According to Bigal et al., opiates and barbiturates are linked to a twofold increase in migraine progression. Triptans, on the other hand, increase the risk of chronic headaches in people who have a high baseline frequency of migraine. Anti-inflammatory medications, such as NSAIDs, were protective in those who had a lower frequency of headache (less than 10 days), but, like triptans, they were associated with an increased risk of migraine progression in those who had a high frequency of headache at baseline (more than 10 days). The study concludes that, while different medication classes are associated with MOH, high headache frequency at baseline is a major predictor of chronic migraine, regardless of medication history (20). This is in line with a systematic review analysis of 29 studies that found ergotamine and triptan-containing drugs to be more favorable when compared to opioids (21).

Multiple psychiatric disorders, including anxiety, mood disorders, depression, and obsessive-compulsive disorder, are associated with MOH, but the extent of their relationship is unclear. Furthermore, according to other studies, 40% of MOH patients met the criteria for depression, and 58% met the criteria for anxiety (22, 23). Substance-related disorders are also another determinant risk factor for the development of MOH, mainly due to the overlap of pathophysiological mechanisms between these two entities as determined by different neurobiological and pharmacological studies (24).

According to Cevoli et al., around 30% of patients with MOH have a family history of chronic headaches with medication overuse (*P* = .0014) and around 22% for substance abuse (*P* = .002). The study further states that relatives of patients with MOH were more likely to suffer from chronic headaches, drug overuse, and substance abuse (25).

Just like other types of headaches, MOH is associated with low income and a low educational level, but it is unclear whether this is the cause or result of the headache (2, 10).

Patients with MOH tend to be active smokers. Also, a body mass index of ≥30 is strongly associated with MOH patients compared to the control group, according to Straube et al. (9).

## Pathophysiology

The precise pathophysiology of MOH remains uncertain. However, research shows that central sensitization has a major role (26, 27). An expansion of the receptive nociceptive field, a decreased nociceptive threshold, and decreased noxious inhibitory control have been reported following chronic analgesic use (28, 29).

The frequent use of headache abortive medications is also thought to deplete 5-hydroxytryptamine (5-HT) in the central nervous system (CNS). This depletion leads to hyperexcitability in the cerebral cortex, potentially causing cortical spreading depression, as well as in the trigeminal system, which leads to increased sensitivity in both peripheral and central pain pathways. Reduced 5-HT levels also cause the trigeminal ganglia to release calcitonin gene-related peptide (CGRP), a peptide involved in pain transmission, which contributes to the sensitization of nociceptive trigeminal neurons (6, 30). Upregulation of other vaso-active and pro-inflammatory mediators such as substance *P* and nitric oxide synthase was also found in the trigeminal ganglia (31).

## Clinical features and comorbidities

MOH is a chronic daily headache that occurs 15 or more days per month as a result of a patient's regular overuse of headache medications (32, 33). This headache could be migraine- or tension-type in nature, and it could be more frequent and severe than the pre-existing headache disorder. However, this is not always the case. MOH frequently shares traits with other forms of chronic daily headaches. There are no specific headache traits connected to MOH. As a result, the presence of a typical clinical presentation, if present, should not discourage doctors from considering secondary headaches and their workup (6). MOH typically occurs after primary headaches, but some studies suggest that it can also occur after secondary headaches (34–37).

There is an association between MOH and other conditions. There may be more to this association than coincidence, which could point to similar pathophysiologic or etiologic processes (6). In addition to psychiatric disorders, there are also behavioral, autonomic, and other comorbidities. Generally, patients with MOH may present with a change in the character of the underlying primary or secondary headache and may exhibit symptoms of some comorbidities associated with MOH.

MOH is linked to biochemical, structural, and functional changes in the brain (38). Among patients with MOH, psychiatric comorbidities are very prevalent. This association could be indicative of a potential causal role (39–41). Examples include abnormal personality traits, especially neuroticism, as well as OCD, depression, anxiety, and sleep disorders like insomnia (18, 22, 23, 39, 40, 42–44). Given this association, conducting a psychological assessment is a crucial first step in evaluating and treating patients with MOH (44). By properly treating the MOH, these psychiatric conditions can also be mitigated (23).

Additionally, behavioral factors are worth taking into account. A pathophysiologic mechanism for the development of MOH in patients with another primary headache may involve dependency-related behavior (24). This does not imply, however, that substance addicts and MOH patients have similar personality traits (45). Furthermore, patients with MOH may behave in ways that resemble those of people who are dependent on other substances. For instance, substance dependence behaviors that can be mentioned include self-medication, anxiety related to receiving prescriptions for drugs, and loss of control over the use of painkillers (33). Other behavioral factors linked to an increased risk of MOH development include smoking, caffeine use, and physical inactivity (19, 42).

Autonomic symptoms can occur in MOH as a result of chronic painkiller stimulation and withdrawal, which can disrupt the balance of neurotransmitters and hormones in the brain and body. They may also be caused by an underlying headache disorder, such as a migraine or cluster headache, which is exacerbated by medication overuse. Some common autonomic symptoms that can accompany a headache include a runny nose, tears, nausea, vomiting, and diarrhea (46).

Other preceding complaints, such as musculoskeletal and gastrointestinal ones, may also be present in MOH patients (18, 47). Additionally, there are notable associations with metabolic disorders like hypertension and obesity (42, 48).

## Diagnosis

Clinicians should always suspect medication overuse headache in a patient who reports frequent headaches, especially if the patient has a history of migraine. It is important to note that the headaches caused by medication overuse have no distinguishing characteristics (49).

The International Classification of Headache Disorders, 3rd Edition Beta (ICHD-3) diagnostic criteria for MOH are as follows;

(1) Headache that occurs 15 or more days per month in a patient who already has a headache disorder.
(2) Overuse of one or more drugs that can be used to treat acute and/or symptomatic headaches for more than 3 months:
  (a) Regular intake of ergotamines, triptans, opioids, or combination analgesics for 10 days per month for more than 3 months, or any combination of ergotamines, triptans, simple analgesics, nonsteroidal anti-inflammatory drugs (NSAID), and/or opioids without overuse of any single drug or drug class alone, or when the pattern of overuse cannot be reliably established.
  (b) Simple analgesics (i.e., acetaminophen, aspirin, or NSAIDs) taken on a regular basis for 15 or more days per month for more than 3 months.
(3) Not better explained by another ICHD-3 diagnosis.

Patients who meet both MOH and chronic migraine criteria are given both diagnoses (50). A high frequency of drug use does not imply that MOH is the only headache disorder present (51). A patient with MOH typically has an underlying primary headache disorder, such as migraine or tension-type headache, that has gradually or abruptly increased in frequency, associated with increased analgesic intake, and eventually, MOH superimposes itself upon the primary headache disorder. Patients may find themselves taking analgesics on a daily or frequent basis at this point, simply to avoid a crippling analgesic withdrawal headache (51). It is important to note that medication overuse can occur not only as a result of treating headaches, but also due to various other factors. While it is not a common occurrence, individuals with existing headache disorders may consume analgesic medications for reasons other than addressing actual headache symptoms, leading to the development of MOH. To illustrate this, patients may take analgesics even in the absence of pain due to behaviors such as ritualistic drug administration or psychological attachment to the medication (52). Moreover, frequent use of analgesics for alternative purposes, such as relieving musculoskeletal pain, can also contribute to the development of MOH in patients with pre-existing headache disorders (46, 53).

The new appendix criteria for a broader concept of chronic migraine from the International Headache Society no longer require headache resolution or a return to the previous headache pattern to confirm the diagnosis of MOH.

MOH can be subdivided into simple (Type I) and complex (Type II). Complex cases may involve long-term use of daily opioids or combination analgesics, multiple medication sources from different prescribers, multiple psychiatric comorbidities, and/or a history of relapse (54).

## Management

There is no global management agreement on medication overuse headaches. However, medication withdrawal is by far the most common treatment (2). Patient education, the use of rescue drugs, and the use of bridging medications are all important throughout the withdrawal period, depending on the severity of the symptoms. When treating the underlying chronic headache and initiating prophylactic medication, the type and intensity of the headache must be considered (55).

Depending on the abused medicine, withdrawal symptoms such as increased headache, vomiting, restlessness, and anxiety can last from 2 days to a month (55). Withdrawal symptoms due to analgesics typically persist for a maximum of 10 days, while the withdrawal symptoms of triptans and ergotamine can last up to 4 and 7 days, respectively. Withdrawal symptoms from certain medications can last up to a month. Simple analgesics, ergotamines, and triptans may be abruptly discontinued without tapering (2). Tapered withdrawal and inpatient monitoring may be required for opioids, barbiturates, and benzodiazepines (55). Inpatient monitoring may also be required for psychological issues, severe comorbidities, and severe withdrawal symptoms. Generally, drug withdrawal and detoxification will improve headache intensity and frequency (55). There is no difference in effectiveness or long-term prognosis between outpatient and inpatient withdrawal detoxification regimens, and outpatient programs are recommended (56).

Patient counseling and advice on drug withdrawal, as well as the effects and consequences of medication overuse, should be addressed. Most patients are unaware that overuse causes headache aggravation and reduces the efficacy of overused drugs for headache relief (2). Sometimes simple counseling is all that is required to manage MOH, resulting in a considerable reduction in medication days and the headache index (57). It is successful in simple MOH cases and effective in most complicated cases (58, 59). Patients should also be informed about withdrawal symptoms during the detoxification period, as well as the possibility of using short-term bridging medication different from the overused medication to relieve withdrawal symptoms based on necessity (2).

The use of rescue and short-term bridging drugs is also useful, depending on the severity of withdrawal symptoms. Medications that have been overused should be avoided during this period (2). For severe withdrawal headaches, there are medications such as NSAIDs, steroids, and triptans that can be used for 7–10 days. Tapering corticosteroids (at least 60 mg of prednisone) and amitriptyline (up to 50 mg) may be beneficial in the treatment of withdrawal symptoms (55). Depending on the severity, antiemetics, tranquilizers, and neuroleptics may be utilized.

Treatment with either lifestyle changes or long-term prophylaxis is important. Some underlying headaches benefit from cognitive behavioral therapy and relaxation approaches. Prophylactic drugs are prescribed based on the type of headache and are given to individuals who do not benefit from withdrawal therapy alone and are having trouble stopping the medication. Rather than starting prophylactic drugs immediately, it is recommended to start them after watching the initial withdrawal treatment (2). Recent studies also suggest that those with primary migraine/tension headaches who also suffer headaches from medication overuse may benefit from beginning preventive prophylactic medication at the beginning of withdrawal therapy to return to an episodic pattern (60). Topiramate at doses up to 200 mg is the only medicine with moderate evidence for preventative treatment of chronic migraine and medication overuse patients (54). Prophylactic medications for primary headaches, such as beta-blockers for migraines or amitriptyline for tension type headaches, should be tried as well (61). Prophylactic therapy for primary headaches relieves overuse headaches and returns the headache pattern to an episodic pattern (47). Currently, new prophylactic drugs are becoming available. CGRP receptor antagonists and monoclonal antibodies have been licensed for migraine prevention. There has been debate about the effectiveness of using botox for preventive treatment (62, 63). Because of the scarcity and high cost of CGRP receptor antagonists and monoclonal antibodies, most primary care patients may benefit from using simple withdrawal procedures to manage medication overuse headaches.

Regardless of the treatment approach, there is a 20%–40% relapse rate within the first year after withdrawal (2) and a 14%–41% recurrence rate after 1 year (55). The use of a combination analgesic following withdrawal therapy, as well as the reintroduction of prior acute medication, all enhance the chance of MOH recurrence. Follow-up therapy with a primary care physician is essential to reduce the risk of relapse. Primary care patient education is a good setting for MOH prevention. Patients should be warned about the risks of improper painkiller use at the time of presentation and during follow-up. Brochures and public awareness by pharmacists and society are essential (2).

## Prevention

Given the high prevalence of migraine and tension-type headaches and the fact that virtually anyone with such a disorder may be at risk of developing MOH, the number of people at risk is substantial. Educational strategies to increase knowledge among health care providers to identify patients at risk, inform their patients about the risks, and modify their headache management habits, information campaigns to raise public awareness, and warning labels on over-the-counter analgesics are possible preventive strategies. As specific modifiable risk factors for MOH may exist, other measures such as restrictions on the use of tranquilizers and tobacco and increased physical activity may also be effective (18).

In general, MOH prevention can be either primary or secondary. Primary prevention techniques are critical for preventing MOH from occurring in the first place. Identifying patients at risk and educating them on the risk of MOH and recommended headache management strategies, optimizing abortive and preventive management of patients with chronic headache disorders, and addressing modifiable risk factors such as smoking, physical inactivity, and tranquilizer use are some examples (64). Secondary prevention techniques are critical for detecting the condition early and halting its progression. This can be accomplished by limiting headache symptomatic medication use to no more than 2 days per week and avoiding previously overused medication classes (65, 66). The severity-dependent scale (SDS) has also been shown to predict medication overuse in chronic headache patients, which is useful for identifying patients who may benefit from detoxification early on. Medication overuse could be predicted with sensitivity, specificity, and positive and negative predictive values of 0.79, 0.84, 0.84, and 0.79 in men and 0.76, 0.77, 0.73, and 0.79 in women. It has also been proposed that increasing the SDS score by one increases the odds ratio (OR) for medication overuse by 1.72. As a result, it was determined that the SDS questionnaire detects medication overuse and dependency-like behavior in people suffering from primary chronic headache (67).

## Conclusion

Medication overuse headache (MOH) is a common and disabling condition that affects millions of people worldwide. It is caused by the frequent or excessive use of painkillers or other drugs for headache relief. MOH can worsen the underlying headache disorder and reduce the effectiveness of preventive treatments. The primary treatment for a drug overuse headache is medication withdrawal. Simple counseling is sometimes all that is necessary to manage MOH. Rescue and bridging medicines are useful depending on the severity of withdrawal symptoms; nevertheless, reuse of overused medication should be avoided. MOH prevention requires prophylaxis and lifestyle adjustments, with topiramate being the sole drug with moderate evidence for preventive therapy. MOH is a preventable and treatable condition that requires awareness, education, and cooperation between patients and health professionals.
